# *Morc2a* p.S87L mutant mice develop peripheral and central neuropathies associated with neuronal DNA damage and apoptosis

**DOI:** 10.1242/dmm.049123

**Published:** 2021-10-25

**Authors:** Geon Seong Lee, Geon Kwak, Ji Hyun Bae, Jeong Pil Han, Soo Hyun Nam, Jeong Hyeon Lee, Sumin Song, Gap-Don Kim, Tae Sub Park, Yang Kyu Choi, Byung-Ok Choi, Su Cheong Yeom

**Affiliations:** 1 Graduate School of International Agricultural Technology and Institute of Green Bio Science and Technology, Seoul National University, 1447 Pyeongchang-Ro, Daewha, Pyeongchang, Kangwon 25354, South Korea; 2 Department of Neurology, Sungkyunkwan University School of Medicine, 81 Irwonr-ro, Gangnam, Seoul 06351, South Korea; 3 Department of Health Science and Technology, SAIHST, Sungkyunkwan University School of Medicine, 81 Irwonr-ro, Gangnam, Seoul 06351, South Korea; 4 Department of Laboratory Animal Medicine, College of Veterinary Medicine, Konkuk University, 120 Nueungdong-ro, Gwangjin, Seoul 05029, South Korea; 5 Stem Cell and Regenerative Medicine Institute, Samgsung Medical Center, Seoul 06351, South Korea; 6 WCU Biomodulation Major, Department of Agricultural Biotechnology, Seoul National University, 1 Gwanak-ro, Gwanank, Seoul 08826, South Korea

**Keywords:** Neuronal apoptosis, CMT2Z, DIGFAN, *Morc2a*, S87L

## Abstract

The microrchidia (MORC)-family CW-type zinc finger 2 (*MORC2*) gene is related to DNA repair, adipogenesis and epigenetic silencing via the human silencing hub (HUSH) complex. *MORC2* missense mutation is known to cause peripheral neuropathy of Charcot-Marie-Tooth disease type 2 Z (CMT2Z). However, there have been reports of peripheral and central neuropathy in patients, and the disease has been co-categorized with developmental delay, impaired growth, dysmorphic facies and axonal neuropathy (DIGFAN). The etiology of *MORC2* mutation-mediated neuropathy remains uncertain. Here, we established and analyzed *Morc2a* p.S87L mutant mice. *Morc2a* p.S87L mice displayed the clinical symptoms expected in human CMT2Z patients, such as axonal neuropathy and skeletal muscle weakness. Notably, we observed severe central neuropathy with cerebella ataxia, cognition disorder and motor neuron degeneration in the spinal cord, and this seemed to be evidence of DIGFAN. *Morc2a* p.S87L mice exhibited an accumulation of DNA damage in neuronal cells, followed by p53/cytochrome c/caspase 9/caspase 3-mediated apoptosis. This study presents a new mouse model of CMT2Z and DIGFAN with a *Morc2a* p.S87L mutation. We suggest that neuronal apoptosis is a possible target for therapeutic approach in *MORC2* missense mutation.

This article has an associated First Person interview with the first author of the paper.

## Abstract (precis)

**Summary:** We present a new mouse model of Charcot-Marie-Tooth disease type 2 Z and developmental delay, impaired growth, dysmorphic facies and axonal neuropathy syndrome with a *Morc2a* p.S87L mutation, exhibiting peripheral and central neuronal apoptosis.

## INTRODUCTION

Neuropathy is caused by nerve damage and leads to symptoms such as weakness, numbness or pain. Charcot-Marie-Tooth disease (CMT) is a hereditary motor and sensory neuropathy commonly inherited as peripheral neuropathy (Tazir et al., 2014). Patients with CMT generally develop distal muscle weakness and atrophy, distal sensory loss, weak ankle dorsiflexion, depressed tendon reflexes and high-arched feet (McDonald, 2012). There is no fundamental therapy for this condition, and treatment is limited to symptomatic approaches, such as surgery, physical and occupational therapy (Mathis et al., 2015).

CMT is caused by mutations in protein-coding genes that play a role in myelin structure, mitochondrial function, cellular trafficking enzymes and amino-acyl tRNAs. CMT type 2 is less common than CMT type 1 and is divided into various subtypes based on the specific genes that are mutated. The recently reported CMT type 2 Z (CMT2Z) is an autosomal-dominant peripheral neuropathy that is caused by a mutation in the microrchidia (MORC)-family CW-type zinc finger 2 (*MORC2*) gene (Sevilla et al., 2016). *MORC2* was first discovered during the sequencing of human brain cDNA and consists of a GHKL (Gyrase B, Hsp90 and MutL)-type N-terminal ATPase domain, a three-stranded coiled-coil domain, a CW domain, and a two-stranded coiled-coil domain near the C terminus (Nagase et al., 1998; Perry and Zhao, 2003; Wang et al., 2010). *MORC2* functions as a transcriptional repressor when overexpressed and leads to the development of several phenotypes through interaction with other genes (Wang et al., 2010). Moreover, *MORC2* has several functions, such as DNA repair by interaction with p21-activated kinase 1 (Li et al., 2012), adipogenesis and lipid homeostasis via ATP citrate lyase phosphorylation and activation (Sanchez-Solana et al., 2014), and epigenetic silencing via interaction with the HUSH complex (Tchasovnikarova et al., 2017).

*MORC2* missense mutation, such as with p.S87L, p.R132L, p.E236G, p.R252W, p.Q400R, p.A4006V, p.C407T, p.T424R, p.A432V and p.D466N, causes neuropathy. Moreover, p.R252W is the mutation most frequently observed, and p.S87L leads to a more severe clinical phenotype (Sevilla et al., 2016). Although CMT is known as a disease associated with peripheral neuropathy, *MORC2* mutations cause clinical symptoms such as severe axonal polyneuropathy, spinal muscular atrophy, cerebellar ataxia, diaphragmatic paralysis, craniofacial dysmorphism and nocturnal hypoventilation (Guillen Sacoto et al., 2020; Schottmann et al., 2016; Zanni et al., 2017). Thus, *MORC2* missense mutation might need to be categorized as new neuropathy rather than solely CMT2Z. *MORC2* missense is also considered a causing factor of developmental delay, impaired growth, dysmorphic facies and axonal neuropathy (DIGFAN; OMIM 619090), and CMT2Z and DIGFAN often overlap in their clinical symptoms (Guillen Sacoto et al., 2020).

There is enormous genetic heterogeneity in the neuropathy-causal single-nucleotide polymorphism (SNP) and difficulties translating preclinical studies from animal and cellular models to a human clinical trial. The co-existence of peripheral and central neuropathy in *MORC2* missense mutation is interesting. However, the etiology of *MORC* mutation-mediated neuropathy remains uncertain. The mouse ortholog of human *MORC2* is microrchidia 2A (*Morc2a*), and it has lethal properties (https://dmdd.org.uk/mutants/Morc2a). To study the mechanism and therapeutic approaches for *MORC2*-mediated neuropathy, *in vivo* studies using appropriate animal models are essential.

Although genetically engineered mouse models of CMT have been developed, no mice with p.S87L have been developed. Because the *MORC2* p.S87L missense mutation leads to severe neuropathy in humans, we selected p.S87L as a target mutation in the design of our mouse model. This study established *Morc2a* p.S87L mutant mice and analyzed their relevance to human peripheral and central neuropathy. CRISPR/Cas9-mediated genome editing was applied, and the founder mice were subjected to further phenotypic and mechanistic studies of peripheral and central neuropathy.

## RESULTS

### Generation of *Morc2a* p.S87L mice by single-stranded template repair

**Fig. 1.**
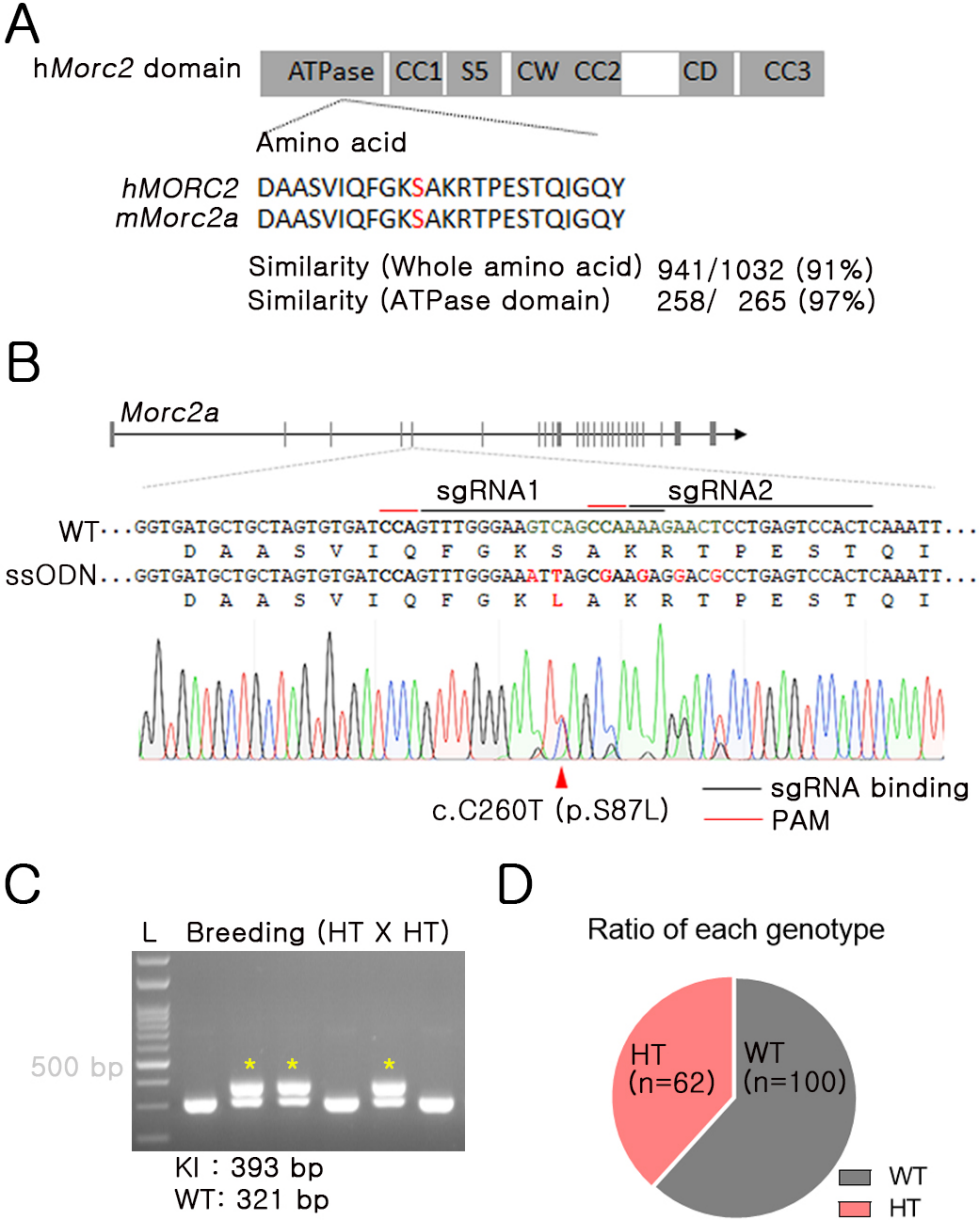
**Generation of B6.*Morc2a* S87L/+ mice.** (A) Domain structure of the human MORC-family CW-type zinc finger 2 (*MORC2*) gene. Red letters indicate the 87th serine in the human MORC2 (hMORC2) and mouse Morc2a (mMorc2a) proteins. CC, coiled-coil domain; CD, chromo-like domain; CW, CW-type zinc finger domain; S5, ribosomal protein S5-like domain. The similarity of amino acids between h*MORC2* (NP_001290185) and m*Morc2a* (NP_001152760) was calculated. (B) Targeted gene editing was conducted using electroporation of the Cas9 protein, single-guide RNA (sgRNA) and single-stranded oligodeoxynucleotide (ssODN) template into one-cell-stage embryos. Red letters indicate the sequences used for nucleotide alteration and amino acids. PAM, protospacer adjacent motif. (C) PCR-based genotyping. A representative image is displayed. Yellow asterisks indicate mouse harboring p.S87L. HT, heterozygote; KI, knock-in; L, ladder; WT, wild type. (D) The ratio of each genotype was calculated after PCR genotyping. Mice were bred using the breeding scheme ‘heterozygote × heterozygote’.

The mouse *Morc2a* gene contains ATPase, CC1, S5, CW, CC2, CD and CC3 domains (Tchasovnikarova et al., 2017), and has ∼91% amino acid similarity to the human *MORC2* gene. Notably, mouse *Morc2a* and human *MORC2* genes exhibit ∼97% amino acid similarity in the ATPase domain (Fig. 1A). Considering that mutation in the ATPase module of *MORC2* causes neurodevelopmental disorder (Guillen Sacoto et al., 2020), the high similarity of the ATPase domain is necessary to develop neuropathy in mice. To produce mice with the p.S87L mutation, CRISPR/Cas9 and single-stranded oligodeoxynucleotides (ssODNs) were applied to mouse embryos. Briefly, two single-guide RNAs (sgRNAs) with overlapping binding sites were used to induce small-sized double-stranded DNA breakage (Jang et al., 2018), and ssODNs with the target (p.S87L) and a silencing mutation were applied together (Fig. 1B). After confirming knock-in by sequencing, founder mice were subjected to heterozygote × heterozygote breeding. No homozygous mice were obtained, which seemed to be caused by the embryonic-lethal characteristic by a neurological developmental abnormality (https://dmdd.org.uk/mutants/Morc2a). Theoretically, the incidence ratio of wild-type (WT) and heterozygote mutant animals should be 1:2 under breeding between heterozygotes. Interestingly, there was a higher frequency of WT versus B6.*Morc2a* S87L/+ mice (62/100) (Fig. 1C,D). Thus, these results suggest the sublethal characteristics of B6.*Morc2a* S87L/+ during embryonic development. According to the International Mouse Phenotyping Consortium database, homozygote *Morc2a* knockout mice exhibited preweaning lethality similar to B6.*Morc2a* S87L/+, but heterozygote *Morc2a* knockout mice did not exhibit sublethality (https://www.mousephenotype.org/data/genes/MGI:1921772). In this study, B6.Morc2a S87L/+ and their WT littermates were used.

### *Morc2a* p.S87L mice exhibit peripheral motor and sensory neuropathy

**Fig. 2.**
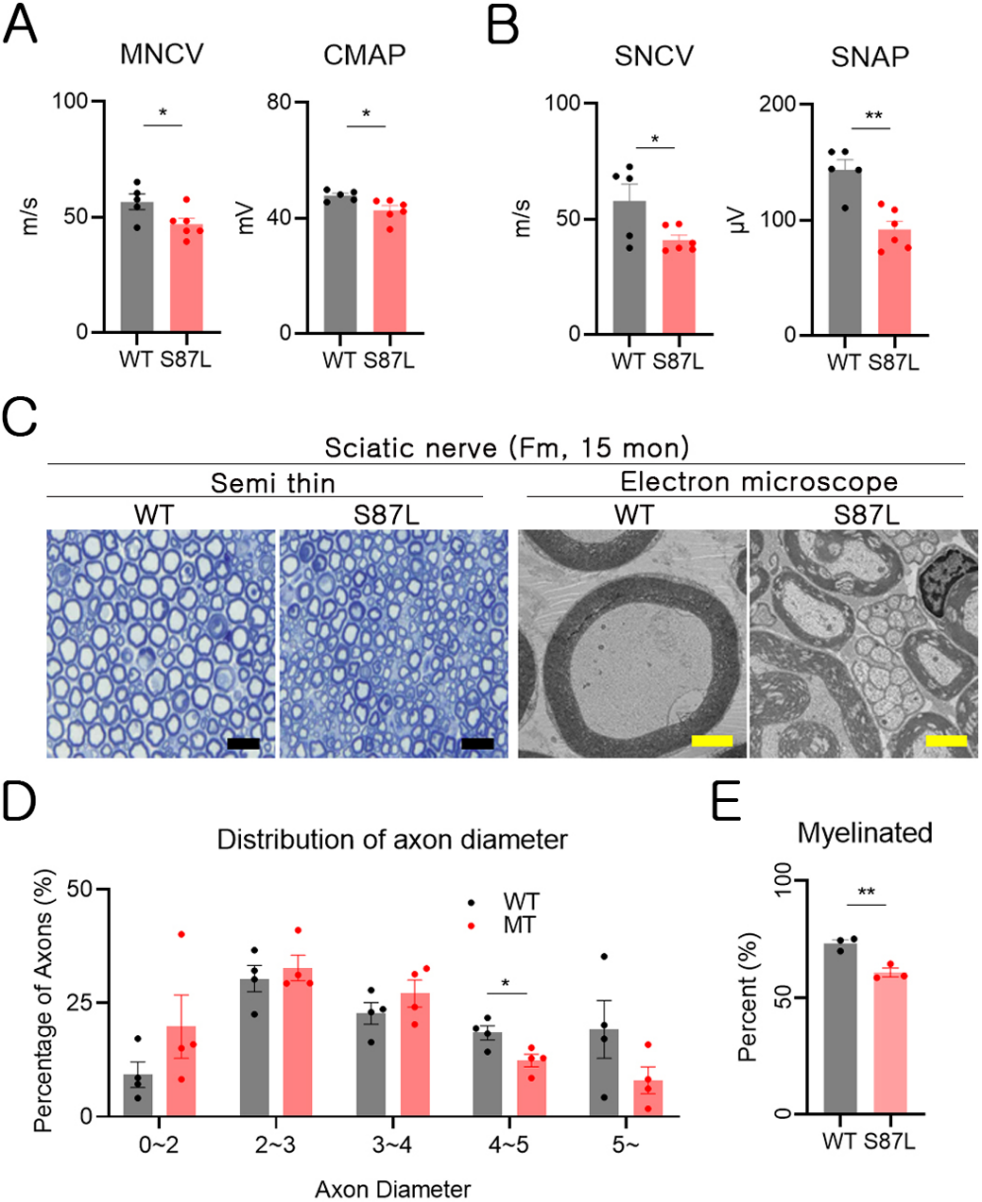
**Electrophysiological and histological analyses of B6.*Morc2a* S87L/+ mice.** (A,B) Motor nerve conducting velocity (MNCV; A, left), compound muscle action potential (CMAP; A, right), sensory nerve conduction velocity (SNVC; B, left) and sensory nerve action potential (SNAP; B, right) were measured in 15-month-old female mice (WT, *n*=5; S87L, *n*=6). (C) Representative semi-thin cross-section images stained with Toluidine Blue (left) and ultra-thin electron micrographs from the same sections (right) from the sciatic nerves of WT and B6.*Morc2a* S87L/+ female mice at 15 months. Scale bars: 20 μm (semi-thin sections) and 2 μm (ultra-thin sections). (D,E) Neuroanatomy was analyzed according to genotype and nerve diameter (D) and myelination of axons (E). Dots indicates data from each mouse sample, and data are displayed as the mean±s.e.m. (WT and S87L, *n*=3 or 4 females at 15 months). *P<0.05, **P<0.01 (unpaired Student's *t*-test).

Motor nerve conduction velocity (MNCV) is the conduction velocity at which electrical signals are transmitted through the motor nerve, and compound muscle action potential (CMAP) is the action potential recorded in the muscle after motor nerve stimulation. The analysis of the electrophysiological profiles of the two groups revealed a significant reduction in MNCV and CMAP in B6.*Morc2a* S87L/+ compared with WT mice, which correlated with the distal lower-limb weakness and peripheral motor neuropathy (Fig. 2A). Sensory nerve conduction velocity (SNCV) is the sensory nerve conduction speed at which electrical signals are transmitted through the sensory nerve, and sensory nerve action potential (SNAP) is the action potential recorded in the sensory nerve. In B6.*Morc2a* S87L/+ mice, SNCV and SNAP were also significantly reduced compared with WT mice (Fig. 2B). The SNAP amplitude is decreased and the SNCV is slowed concomitant with the sensory impairment in human patients with *MORC2* mutation (Albulym et al., 2016; Hyun et al., 2016; Sevilla et al., 2016). Therefore, these results demonstrated that the B6.*Morc2a* S87L/+ mouse is suitable as a model of hereditary motor and sensory peripheral neuropathy.

### *Morc2a* p.S87L mice develop axonal neuropathy

The integrity of peripheral nerves was assessed histologically using semi-thin sections of the sciatic nerve. B6.*Morc2a* S87L/+ mice exhibited a lower number of large myelinated fibers in the sciatic nerve than WT mice, as assessed using Toluidine Blue staining. Moreover, an electron microscopic examination revealed a pronounced depletion of myelinated fibers in the axons of B6.*Morc2a* S87/L mice compared with WT mice, and some myelin sheaths appeared to be disproportionally thinly myelinated (Fig. 2C). The analysis of the distribution of axon diameters showed a predominant loss of large-diameter fibers of all diameters with relative preservation of, or increase in, small fibers (Fig. 2D), which is a very similar anatomical feature to that of patients with CMT2Z (Hyun et al., 2016). We calculated the amount of myelinated and unmyelinated axons and found that the sciatic nerves of B6.*Morc2a* S87L/+ mice exhibited a significant decrease in myelinated axons (Fig. 2E). Because 15-month-old mice were utilized in this experiment, this severe demyelination represents the end stage of axonal neuropathy. Thus, 4-month-old mice were analyzed to confirm the early onset of neurodegeneration. The Toluidine-Blue-stained and electron microscopic images obtained were similar to those of the 15-month-old B6.*Morc2a* S87L/+ mice (Fig. S1). These findings suggest that B6.*Morc2a* S87L/+ mice developed axonal neuropathy under 4 months of age.

### The *Morc2a* p.S87L mutation causes locomotive dysfunction and skeletal muscle weakness

**Fig. 3.**
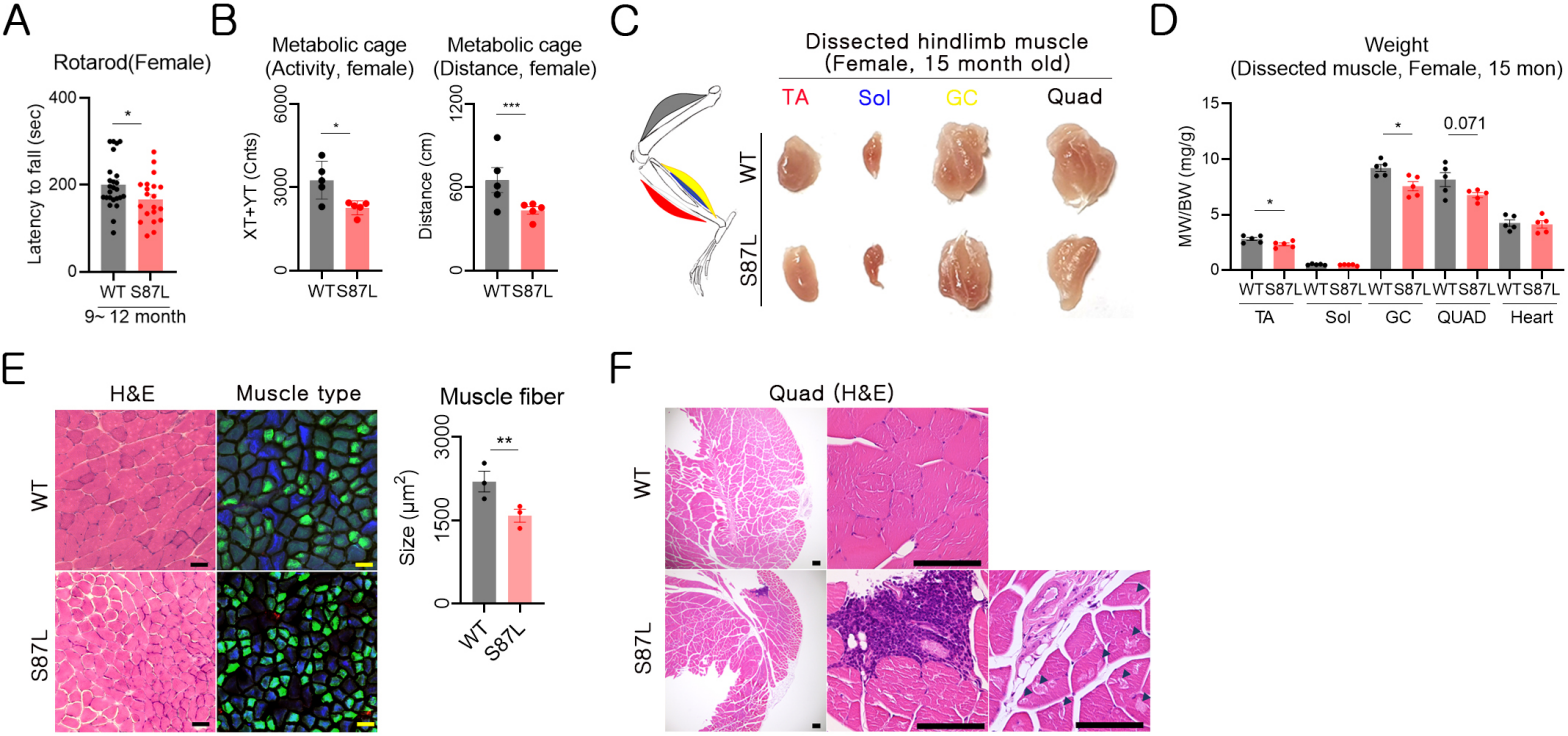
**Distal muscular atrophy and weakness in the B6.*Morc2a*.S87L/+ mice.** (A) Locomotive dysfunction was evaluated in female mice using the rotarod test over a span of 12 months (WT, *n*=5; S87L, *n*=4). Latency to fall off the rotarod was recorded in the accelerating (4-45 rpm) condition. Each dot represents the data collected over 3 months (mean±s.e.m.). *P<0.05 (unpaired Student's *t*-test). (B) Activity (left) and distance of movement (right) were analyzed for 2 days in 15-month-old female mice (WT, *n*=5; S87L, *n*=5). The data were collected for 48 h from a single mouse and are presented as the mean±s.e.m. Each dot represents data from individual mice. *P<0.05, ***P<0.001 (unpaired Student's *t*-test). (C) Representative images of dissected muscle from WT and B6.*Morc2a*.S87L mice with a similar weight. GC, gastrocnemius; Quad, quadriceps femoris; Sol, soleus; TA, tibialis anterior. The abbreviations are shown in the same color text as the respective muscle in the diagram on the left. (D) Weights of the same muscle types from the left and right legs were measured, and average values are displayed (WT, *n*=5; S87L, *n*=5). Data are presented as the mean±s.e.m. *P<0.05 (unpaired Student's *t*-test). (E) Analysis of muscular atrophy using the TA muscle from WT and B6.*Morc2a* S87L/+ mice. The TA muscle was stained with H&E, and immunohistochemistry for myosin-2 (green) and myosin-4 (blue) was performed (left). The size of muscle fibers (200 fibers per mouse) was measured, and average values were analyzed (right) (WT, *n*=5; S87L, *n*=5). **P<0.01 (unpaired Student’s *t*-test). (F) Pathohistological analysis of the Quad muscle of WT and B6.*Morc2a* S87L/+ mice. Scale bars: 100 μm.

Patients with *MORC2*-mediated CMT2Z display severe distal muscle weakness (Fenton et al., 2020; Sevilla et al., 2016); thus, locomotive dysfunction was assessed using the rotarod apparatus for 12 months. Female B6.*Morc2a* S87L/+ animals exhibited a significant decrease in the accelerated rotarod condition compared with WT animals at 6-9 months of age and a relatively reduced fall time in the constant rotarod condition. In contrast, male B6.*Morc2a* S87L/+ mice showed a somewhat higher latency to fall than did their WT counterparts (Fig. 3A; Fig. S2). Rotarod analysis is a method that is used to evaluate balance and motor coordination and can be used for assessing the CMT phenotype. B6.*Morc2a* S87L/+ mice seemed to develop locomotive dysfunction at the age of 6-9 months. Human patients with the *MORC2* p.S87L mutation present an early-onset disease (Hyun et al., 2016; Sevilla et al., 2016); however, B6.*Morc2a* S87L/+ mice showed a late clinical sign. When considering the early onset of the axonal neuropathy in the *Morc2a* p.S87L/+ animals, this mild locomotive dysfunction was not expected, and we hypothesized that the low weight load to the distal limbs caused it. Similar to the decrease in motor activity observed in human patients with CMT (Kennedy et al., 2019), B6.*Morc2a* S87L/+ mice displayed a significant reduction in activity and moving distance (Fig. 3B; Fig. S3).

Notably, the neuropathy detected in B6.*Morc2a* S87L/+ animals caused progressive muscle atrophy, which results in muscular weakness. We analyzed the muscular phenotype by measuring the weight of a representative muscle of the hindlimb. Similar to the muscular atrophy observed in patients with CMT, all skeletal muscles, such as the tibialis anterior (TA), soleus (Sol), gastrocnemius (GC) and quadriceps femoris (Quad) muscles, were smaller in the B6.*Morc2a* S87L/+ mice than in the WT animals. The weight of smooth muscular organs, such as the heart, was similar between WT and B6.*Morc2a* S87L/+ mice (Fig. 3C,D). Histological analysis of the TA muscle revealed that the muscular bundle size was ∼30% smaller in B6.*Morc2a* S87L/+ than in WT animals, which indicated muscular atrophy (Fig. 3E). The Quad muscle exhibited immune cell infiltration and an interesting pathological muscle-fiber destruction, in a different shape from the muscular dystrophy reported previously in CMT (Fig. 3F). There was destruction in the central core of muscle fibers, which was similar to the pathological change from central core disease (Jungbluth, 2007).

### Mice with *Morc2a* p.S87L develop cerebellar ataxia and motor neuron degeneration

**Fig. 4.**
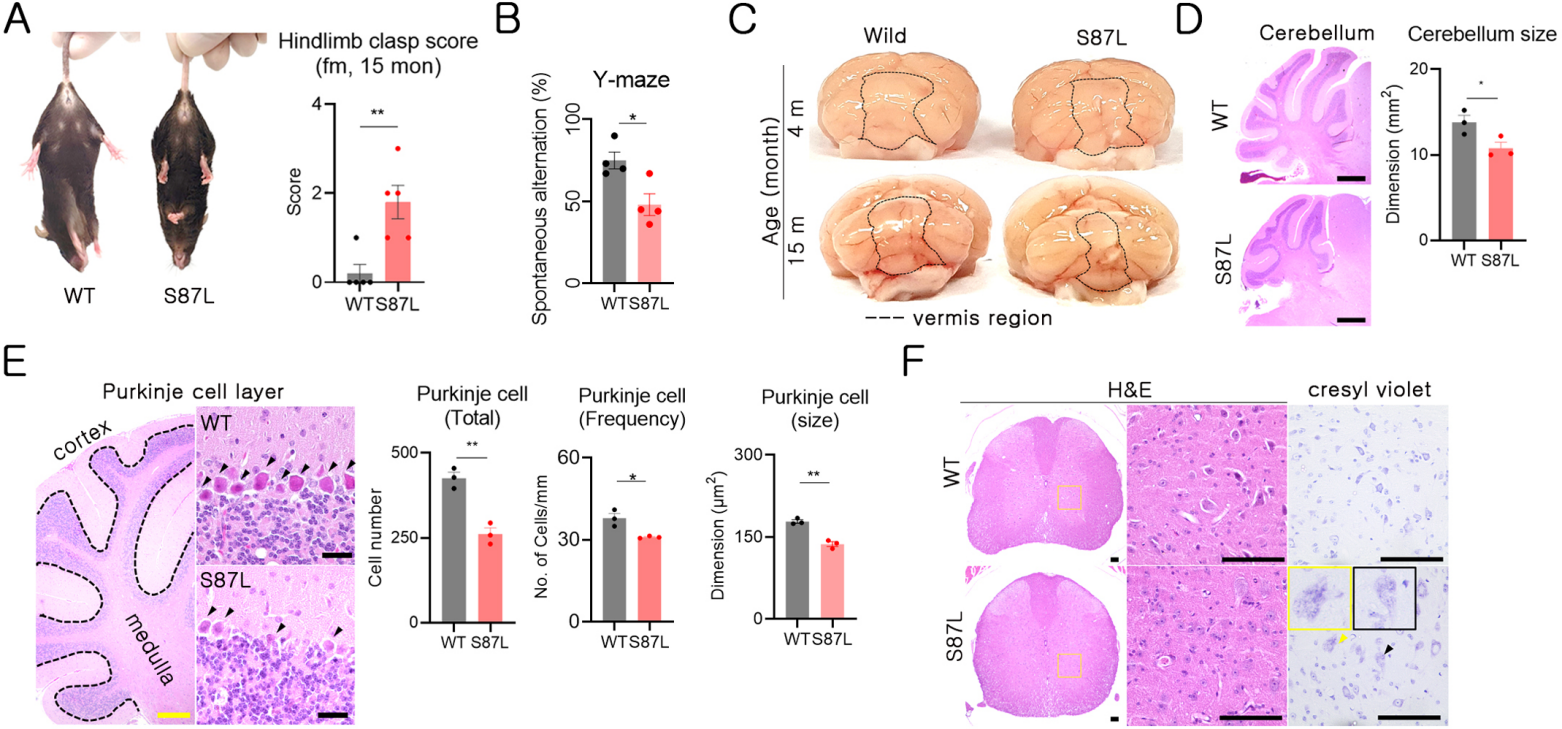
***Morc2a* S87L mice developed cerebella ataxia and spinal cord motor neuron degeneration.** (A) Representative images from WT and B6.*Morc2a*.S87L mice showing hindlimb clasping (left). Hindlimb clasp scores were measured in 15-month-old mice and analyzed (right). Each dot represents data from each mouse (WT, *n*=5; S87L, *n*=5). **P<0.01 (unpaired Student's *t*-test). (B) Cognitive function was assessed in the Y-maze test using 10- to 12-month-old mice. Each dot represents data in percentage of spontaneous alternation returns from each mouse (WT, *n*=4; S87L, *n*=4). *P<0.05 (unpaired Student's *t*-test). (C) Gross morphology of brains from 4-month-old and 15-month-old WT and B6.*Morc2a* S87L mice. (D) Microscopic images of the cerebellum of 15-month-old WT and B6.*Morc2a* S87L mice are displayed (left) (WT, *n*=3; S87L, *n*=3). Scale bars: 1 mm. Data on cerebellum size are presented as the mean±s.e.m. (right). (E) Representative images from the Purkinje cell layer from WT and S87L mice (left). Arrowheads indicate Purkinje cells. Scale bars: 1 mm (yellow) and 100 µm (black). Quantification of the number and size of Purkinje cells in the cerebellum in 15-month-old mice (WT, *n*=3; S87L, *n*=3). In an analysis of total cell number, each dot indicates the total Purkinje cell number from an individual mouse (left graph). In a frequency analysis, the Purkinje cell numbers from 1 mm from seven different regions were calculated, and the average from each mouse was analyzed (middle graph). The size of Purkinje cells was measured using the ImageJ program (right graph). Each dot represents the average size of Purkinje cells in an individual mouse, and 100 cells were analyzed per mouse. *P<0.05, **P<0.01 (unpaired Student's *t*-test). (F) Histological analysis of the cervical spinal cord using H&E and Cresyl Violet. The black and yellow arrowheads indicate motor neuron degeneration. Scale bars: 100 µm.

There are clinical reports of cerebellar ataxia and craniofacial disorder in human patients with *MORC2* gene mutation (Guillen Sacoto et al., 2020; Schottmann et al., 2016; Zanni et al., 2017). Therefore, we utilized the hindlimb clasping test for the behavioral evaluation of central nervous system (CNS) neurodegeneration. B6.*Morc2a* S87L/+ mice showed a significant increase in the hindlimb clasping score compared with WT mice (Fig. 4A). Next, we conducted the Y-maze spontaneous alternative test to evaluate cognitive function. B6.*Morc2a* S87L/+ mice at 10-12 months exhibited a significant decrease in spontaneous alternative percentage compared with WT mice (Fig. 4B). This indicated that B6.*Morc2a* S87L/+ mice also developed cognitive impairment due to central nervous neurodegeneration. Further macroscopic and microscopic analyses revealed the presence of cerebellar atrophy in the B6.*Morc2a* S87L/+ animals; mainly, the size of the vermis was reduced in these mice (Fig. 4C), and the cerebellar atrophy appeared to be primarily caused by degeneration of the whole cerebellar lobe. No significant change was observed in the cerebellar arbor vitae region (Fig. 4D; Fig. S4). Notably, Purkinje cell number and size were decreased significantly in B6.*Morc2a* S87L/+ mice (Fig. 4E). Purkinje cell degeneration is frequently observed in human patients with cerebellar ataxia (Hoxha et al., 2018; Sausbier et al., 2004).

Further histological examination revealed frequent neuronal pathological damage in the cerebral cortex regions located near the hippocampus (Fig. S4), but overall cerebral damage was not severe in 15-month-old B6.*Morc2a* S87L/+ mice. Next, we checked for pathological changes in the spinal cord and found a loss of motor neurons in the anterior horn in B6.*Morc2a* S87L/+ mice. Furthermore, Cresyl Violet staining revealed that motor neurons seemed to be in the process of dissolving (Fig. 4F). To check the progressive pattern of the *Morc2a* p.S87L-associated neuropathy, young mice were used in a histological examination. Although, we observed significant axonal neuropathy in the sciatic nerve from 4-month-old B6.*Morc2a* S87L/+ mice (Fig. S1), the neuron degeneration of the cerebellar and spinal cord was not apparent in mice of the same age (Fig. S5).

### *Morc2a* p.S87L/+ mice exhibit accumulation of DNA damage and apoptosis in neuronal cells

**Fig. 5.**
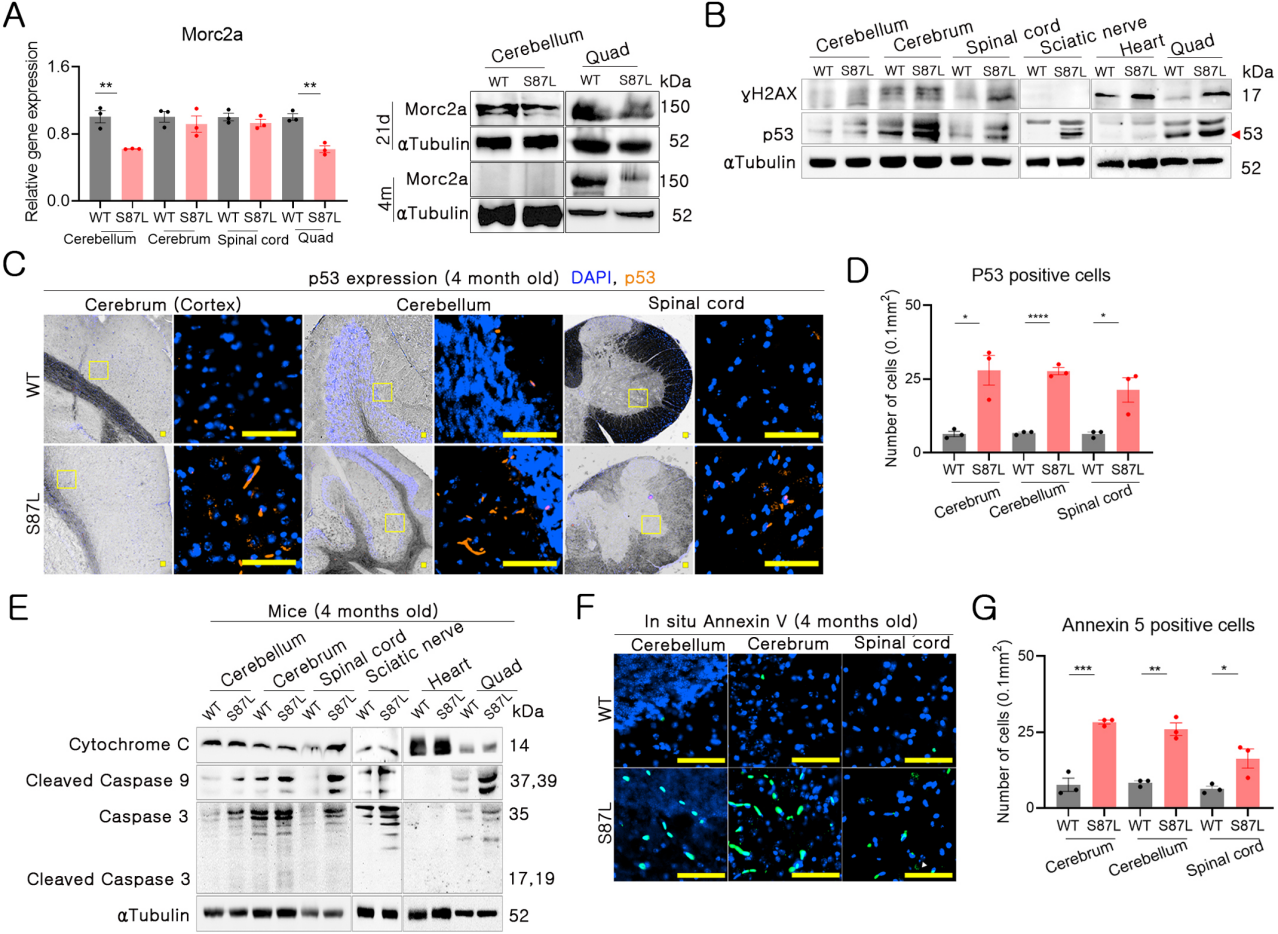
**Accumulation of DNA damage and neuronal apoptosis in *Morc2a* S87L mice.** (A) *Morc2a* gene expression was confirmed by analyzing results from mRNA quantification (tissue from 4-month-old mice) (left) and protein detection (tissue from 21-day-old and 4-month-old mice) (right). Proteins from three different mice were analyzed. (B) Western blotting for p53, γH2AX and tubulin in nervous tissues and muscle from 4-month-old mice. Proteins from three different mice were analyzed. (C,D) Immunofluorescence for p53 in the cerebral cortex, cerebellar Purkinje cells and spinal cord anterior horn region. Scale bars: 200 µm. (D) p53-positive cells were calculated from a 0.1 mm^2^ region of four different areas, and the average number obtained for each mouse from the 4-month-old mice was analyzed (WT, *n*=3; S87L, *n*=3). The dots indicate data from each sample, and data are displayed as the mean±s.e.m. *P<0.05 and ****P<0.00001 (unpaired Student's *t*-test). (E) Apoptosis was confirmed by detecting cytochrome C, cleaved caspase 9 and caspase 3 by western blotting in tissues from 4-month-old mice. Proteins from three different mice were analyzed. (F) Detection of apoptotic cells by immunofluorescence using cerebellum, cerebrum and spinal cord samples from 4-month-old mice. Scale bars: 200 µm. (G) Quantification of Annexin V expression in a 0.1 mm^2^ region of four different areas from 4-month-old mice (WT, *n*=3; S87L, *n*=3). Data are displayed as the mean±s.e.m. *P<0.05, **P<0.01, ***P<0.001 (unpaired Student's *t*-test). Western blotting signal intensity was calculated and is shown in Fig. S6.

Morc2a protein expression in Quad muscle was consistently decreased in 21-day-old and 4-month-old B6. *Morc2a* S87L/+ mice compared with WT mice. In contrast, Morc2a protein expression in the cerebellum was decreased in 21-day-old B6.*Morc2a* S87L/+ mice compared with WT mice but was not detected in 4-month-old mice. In adult mice, Morc2a protein is mainly detected in the testis and skeletal muscle (Shi et al., 2018). *Morc2a* mRNA and Morc2a protein are strongly expressed in the nervous system in the embryonic or neonatal stage, but expression levels progressively decrease with aging (Sancho et al., 2019). B6.*Morc2a* S87L/+ mice seemed to exhibit a decrease in *Morc2a* gene expression in the cerebellum and Quad muscle, with spatiotemporal expression (Fig. 5A).

Because *MORC2* is related to DNA repair, we evaluated early-stage DNA damage by analyzing γH2AX and p53 (also known as TP53) expression in mice. γH2AX seemed to be highly expressed in the spinal cord, heart and Quad muscle of B6.*Morc2a* S87L/+ mice. In addition, p53 was expressed at high level in the brain, spinal cord, sciatic nerve and Quad muscle in these animals, whereas the heart exhibited weak p53 expression. Although the expression pattern of γH2AX and p53 did not exactly match, B6.*Morc2a* S87L/+ mice exhibited overall higher γH2AX and p53 expression than WT mice (Fig. 5B). These results indicated high-frequency DNA damage in B6.*Morc2a* S87L/+ mice. Additional immunofluorescence analysis revealed the significant upregulation of p53 in the neuronal cells of the cerebral cortex and cerebellum and in spinal cord motor neurons in B6.*Morc2a* S87L/+ animals (Fig. 5C,D). Although decreased *Morc2a* expression in 4-month-old B6.*Morc2a* S87L/+ mice was limited to cerebellum and Quad muscle, an increase in γH2AX- and p53-positive cells was confirmed in cerebellum, cerebellum and spinal cord. These results may be due to the different expression patterns of *Morc2a* according to the tissue or age in the nervous system.

DNA damage causes mitochondrial apoptosis, and genes relating to mitochondrial functions can cause neuropathy or ataxia (Higuchi et al., 2018). B6.*Morc2a* S87L/+ mice seemed to develop apoptosis because of a high accumulation of DNA damage. Thus, we tried to confirm apoptosis using traditional western blotting in the primarily affected organ tissues. We observed high cytochrome c expression in the spinal cord. Moreover, we detected activation of caspase 3 and caspase 9 in the cerebellum, cerebrum, spinal cord, sciatic nerve and Quad muscle of B6.*Morc2a* S87L/+ mice (Fig. 5E). This indicates that the CNS neurons of B6.*Morc2a* S87L/+ animals were under cytochrome c/caspase 9/caspase 3-mediated apoptosis. Additionally, Annexin-V (also known as annexin A5) staining revealed significantly more frequent apoptotic neuronal cells in B6.*Morc2a* S87L/+ versus WT animals (Fig. 5F,G).

## DISCUSSION

In the context of genetic disorders, genetically engineered mice may provide new insights into the disease mechanism or clinical symptoms. In this study, we developed B6.*Morc2a* S87L/+ mice, which represent a good CMT2Z and DIGFAN model. They exhibited delayed motor and sensory nerve conduction time, severe axonal neuropathy, muscular weakness, locomotive dysfunction, cerebella ataxia and motor neuron degeneration. Moreover, the *Morc2a* p.S87L mutation seemed to cause higher DNA damage accumulation and consequent neuronal degeneration accompanying apoptosis.

When DNA damage occurs, *MORC2* might regulate chromatin remodeling through ATP hydrolysis (Li et al., 2012). We also observed high-frequency DNA damage and neuronal cell apoptosis in the peripheral and central nervous systems in the B6.*Morc2a* S87L/+ mice. Considering the recently reported neurodevelopment with craniofacial dysmorphism caused by *MORC2* ATPase module mutation (Guillen Sacoto et al., 2020), the peripheral and central neuropathy in *Morc2a* p.S87L seemed to be an expected observation. *De novo MORC2* missense mutation causes epigenetic silencing by the HUSH complex (Douse et al., 2018). Thus, *Morc2a* p.S87L possibly triggers the accumulation of DNA damage. Nevertheless, the causal role of neuronal cell death in CMT2Z and DIGFAN could not be determined clearly. Further investigation by regulating the apoptosis cycle and evaluating consequential peripheral and central phenotypes is needed.

In humans, CMT2Z first affects the peripheral nerves and then progresses to other structures (Sevilla et al., 2016); therefore, neuropathy in the CNS might be unexpected. Nevertheless, B6.*Morc2a* S87L/+ mice showed degeneration of motor neurons of the anterior horn of the spinal cord, cerebral cortex damage, cognitive impairment and cerebellar ataxia accompanying Purkinje cell loss. Patients with CMT2Z with *MORC2* p.T362R mutation develop cerebellar atrophy (Schottmann et al., 2016; Zanni et al., 2017). The human *MORC2* gene has several alternative mRNA forms, and p.T362R is the same as p.T424R and p.T429R. There are several SNPs in the human *MORC2* gene, but spinal muscular atrophy is only reported in patients with the p.T362R (p.T424R) and p.S87L mutations (Hyun et al., 2016; Zanni et al., 2017). The *MORC2* p.S87L and p.T424R mutations change the *MORC2* dimerization module and ATPase activity. This suggests a shared mechanism between the disorders caused by the p.S87L and p.T362R(p.T424R) mutations.

B6*.Morc2a* S87L/+ mice showed cerebral cortex damage and cognitive disorder. In addition, cognitive impairment was identified in human CMT patients of various subtypes (Kasselimis et al., 2020; Pontillo et al., 2020; Werheid et al., 2016). Moreover, CMT2Z patients can develop cognitive impairment (Ando et al., 2017). B6.*Morc2a* S87L/+ mice exhibited a decrease in *Morc2a* gene expression; therefore, the *Morc2a* p.S87L missense mutation seemed to cause neuropathy via loss of function. However, to confirm that the accumulation of apoptotic cells and CNS neuropathy are caused by the *MORC2* S87L mutation, additional clinical data and animal experiments are required.

This study raises the need for further studies on the location and timing of *Morc2a* gene expression, which will be necessary for predicting clinical symptoms and developing therapies for CMT2Z and DIGFAN. However, there is no reported chemical or recombinant protein that can supplement *Morc2a* function. The adeno-associated virus could be a candidate for restoration of *Morc2a* function. However, *MORC2* is related to many cancer types (Pan et al., 2018; Yang et al., 2020; Zhang et al., 2018), so regulating *Morc2a* gene expression is critical. Thus, gene therapy requires a careful approach, and gene correction with *in vivo* and *ex vivo* strategy could be possible.

Here, we present a new mouse model of CMT2Z and DIGFAN with a *Morc2a* p.S87L mutation. These animals developed a critical peripheral and central phenotype similar to that of human patients. We also showed that *Morc2a* mutation causes DNA damage accumulation, especially in neuronal cells, and this is related to neuronal degeneration via apoptosis. The B6.*Morc2a* S87L/+ mice provide new insights into disease mechanisms and may be utilized to investigate CMT and DIGFAN therapies further. In turn, *MORC2* is considered a causative gene of CMT with axonal neuropathy; thus, the diagnosis of central neuropathy was omitted in most clinical diagnoses in these patients. However, our findings suggest that central neuropathy could be considered in patients with *MORC2* missense mutation. There are still no drugs for a fundamental cure of human CMT2Z and DIGFAN, and, based on this study, we suggest that neuronal apoptosis is a possible target for the therapeutic approach.

## MATERIALS AND METHODS

### Generation of B6.*Morc2a* S87L/+ mice

After superovulation, fertilized embryos were collected and used for microinjection into the pronucleus (Micromanipulator System, Eppendorf, Hamburg, Germany). An injection solution for the introduction of the *Morc2a* p.S87L mutation was prepared that contained the SpCas9 mRNA (Toolgen, Seoul, South Korea) (50 ng/µl), sgRNAs (10 ng/µl) and ssODN (50 ng/µl). sgRNAs were synthesized by *in vitro* transcription after PCR amplification (Thermo Fisher Scientific, Waltham, MA, USA), and ssODN was prepared by a commercial synthesis service (Integrated DNA Technologies, Coralville, IA, USA). Detailed information on the sgRNAs and primers used here is provided in Table S1. Embryos were transferred to the oviducts of surrogate mice. The resulting pups were subjected to genotyping using PCR and Sanger sequencing. Next, *Morc2a* p.S87L heterozygous mice (B6.*Morc2a* S87L/+) and their WT littermates were subjected to further CMT phenotyping experiments. This study was approved by the Institutional Animal Care and Use Committee of Seoul National University (SNU-210303-1) and conducted in accordance with Korean Animal Welfare Laws.

### Electrophysiological study

Nerve conduction studies (NCSs) were performed as described previously (Lee et al., 2015). The fur from the distal back to the hind limbs was removed entirely. The NCSs were performed using a Nicolet VikingQuest device (Natus Medical). For the performance of the motor NCS, the stimulating cathodes were placed on the sciatic notch and 6 mm distal to the sciatic notch, while the recording electrodes were placed on the muscle belly of the gastrocnemius muscle. A ground electrode was placed on the animal's back. MNCV and CMAP amplitudes were determined by an independent examiner blinded to the genotypes and treatment groups. We measured CMAPs at supramaximal stimulation. In a sensory NCS, the stimulating cathode was placed on the tail, and the recording electrode was placed on the tail 30 mm proximal to the stimulating cathode. Furthermore, a ground electrode was placed on the animal's back. An independent examiner determined the SNCV and SNAP amplitudes.

### Nerve histological and electron microscopic analysis

The sciatic nerves from WT and B6.*Morc2a* S87L/+ mice were biopsied, and pathological examinations were performed using light and electron microscopy. Specimens were individually fixed in 2% glutaraldehyde in 25 mM cacodylate buffer. Semi-thin sections were stained with Toluidine Blue, and ultra-thin samples were contrasted with uranyl acetate and lead citrate. After incubation for 1 h in 1% OsO_4_, the specimens were dehydrated in an ethanol series, passed through propylene oxide and embedded in epoxy resin (Epon 812, Oken, Nagano, Japan). Three samples per group were analyzed under a light-field microscope. Subsequently, ultra-thin sections (60 nm) were collected on 200 mesh nickel grids and stained for 10 min with 1% uranyl acetate and Reynolds’ lead citrate. The specimens were observed under an HT7700 electron microscope (Hitachi, Tokyo, Japan) at 80 kV. The distribution of myelinated fibers was analyzed by measuring the myelin fiber size using the Zeiss Zen2 program (Carl Zeiss, Oberkochen, Germany). The number of myelinated fibers in a montage consisting of more than 20 photographs taken at 600× magnification using semi-thin sections was evaluated in each mouse.

### Rotarod performance test

Rotarod performance tests were conducted to evaluate the motor and balance abilities of the B6.*Morc2a* S87L/+ mice. After 3 days of adaptation before the initial experiment, two different protocols of accelerating and constant rotation were applied in the rotarod test. In the accelerating condition, the velocity was gradually increased from 4 rpm to 45 rpm over 300 s. In the constant condition, the speed was maintained for 300 s after increasing from 4 rpm to 30 rpm over 60 s. These two rotarod experiments were conducted with a 3-day interval. For each test, mice were subjected to the experiment three times a day with a 10 min break between each measurement. The latency of the fall time was recorded, and an averaged value was used for analysis.

### Moving activity analysis

Moving activity was analyzed based on moving frequency and total distances using a TSE System (TSE Systems, Bad Homburg, Germany). Mice were individually housed in metabolic cages at 23°C with a 12 h light/12 h dark cycle and *ad libitum* access to feed and water. Mice were acclimated for 48 h before data recording, and data acquisition was conducted for an additional 48 h. Briefly, the infrared radiation sensor detected the movements of mice with the *x*- and *y*-axes at 100 Hz every 12 min. The activity phenotype was analyzed using the formula XT (XA+XF)+YT (YA+YF), and distance was calculated using data that measured the whole movement.

### Dissected muscle weight analysis

At 15 months of age, mice were euthanized, and the muscles dissected from both hindlimbs were collected for weight measurement and further analysis. TA, Sol, GC, Quad and heart muscle sampling was conducted according to a protocol reported previously (Shinin et al., 2009).

### Histological analysis [Hematoxylin and Eosin (H&E), Cresyl Violet, immunohistochemistry and Annexin V immunofluorescence]

Whole-mouse perfusion was performed using prewarmed PBS and 4% paraformaldehyde. Subsequently, paraffin blocks were prepared or additional precipitation in 20% sucrose for cryo-fixed block production was performed. For H&E staining, deparaffinized tissues were stained with 0.1% Mayer's H&E solution. For Cresyl Violet staining, cervical sections were stained with 0.1% Cresyl Violet solution (Abcam, Cambridge, UK). Immunohistochemistry was performed using deparaffinized tissues via the procedure of control serum blocking, primary and secondary antibody incubation, and signal detection with 3,3′-diaminobenzidine. Information on the antibodies used in this study is provided in Table S2. *In situ* detection of apoptosis using cryo-fixed tissues was conducted by incubating the sections with an Annexin V-FITC antibody (Trevigen, Gaithersburg, MD, USA) for 1 h at room temperature, followed by signal detection using Cytation 5 (BioTek, Winooski, VT, USA).

### Hindlimb clasping analysis

A mouse was grasped by the front tail and lifted for 10 s, to observe the hindlimb position. The clasping score was calculated using previously reported criteria (Miedel et al., 2017), as follows: 0, the hindlimbs were spread outward from the abdomen; 1, the mouse exhibited normal movement, but one hindlimb showed incomplete splaying and mobility loss; 2, both hind limbs exhibited incomplete swelling and loss of mobility; 3, both hindlimbs were fully contracted with curled toes.

### Y-maze spontaneous alternation test

The Y-maze test was performed in connection to a camera and computer that monitored movement. The Y-maze consisted of three identical arms (35 cm long×20 cm high) that extended from the center at a 120° angle and were labeled as A, B and C. Mice were placed within arm A and allowed to explore for 5 min, the testing period. An arm entry was defined as completely placed four paws in an arm. For each test, the Y-maze was cleaned using 70% ethanol between trials to remove olfactory cues. The sequence of the arm entries was recorded using a VideoMot2 monitoring system (TSE Systems, Chesterfield, MO, USA). Percentage of spontaneous alternation was calculated as follows: the number of actual spontaneous alternations or alternate arm returns/the maximum number of alternations (total number of arm entries −2)×100 (Miedel et al., 2017).

### Western blotting

Each tissue was collected and lysed in RIPA buffer (Intron, Gyeonggi, South Korea) containing proteinase inhibitors for 4 h at 4°C. After SDS−PAGE, proteins were electrotransferred onto polyvinyl fluoride (PVDF) membranes (EMD Millipore, Billerica, MA, USA). The membranes were incubated in a blocking buffer (1× TBS and 5% w/v non-fat milk) for 1 h at room temperature. After 24-48 h of incubation with primary antibodies at 4°C, the membranes were washed with Tris phosphate-buffered saline. The immunoreactive proteins were detected using enhanced chemiluminescence (ECL kit; Abclon, Seoul, South Korea) after horseradish peroxidase-conjugated secondary antibody incubation for 1 h at room temperature. Information on the antibodies used in this study is provided in Table S2.

### Statistical analysis

Statistical analysis was performed with unpaired Student's *t*-test using Prism (Version 9.0, GraphPad Software, San Diego, CA, USA).

## Supplementary Material

Supplementary information
