# Supplementary material for: Morc2a p.S87L mutant mice develop peripheral and central neuropathies associated with neuronal DNA damage and apoptosis
Source: Dis Model Mech. 2021 Oct 25;14(10):dmm049123. doi: 10.1242/dmm.049123 (PMC8560500; doi:10.1242/dmm.049123)
Supplement: Supplementary information [file dmm-14-049123-s1.pdf]

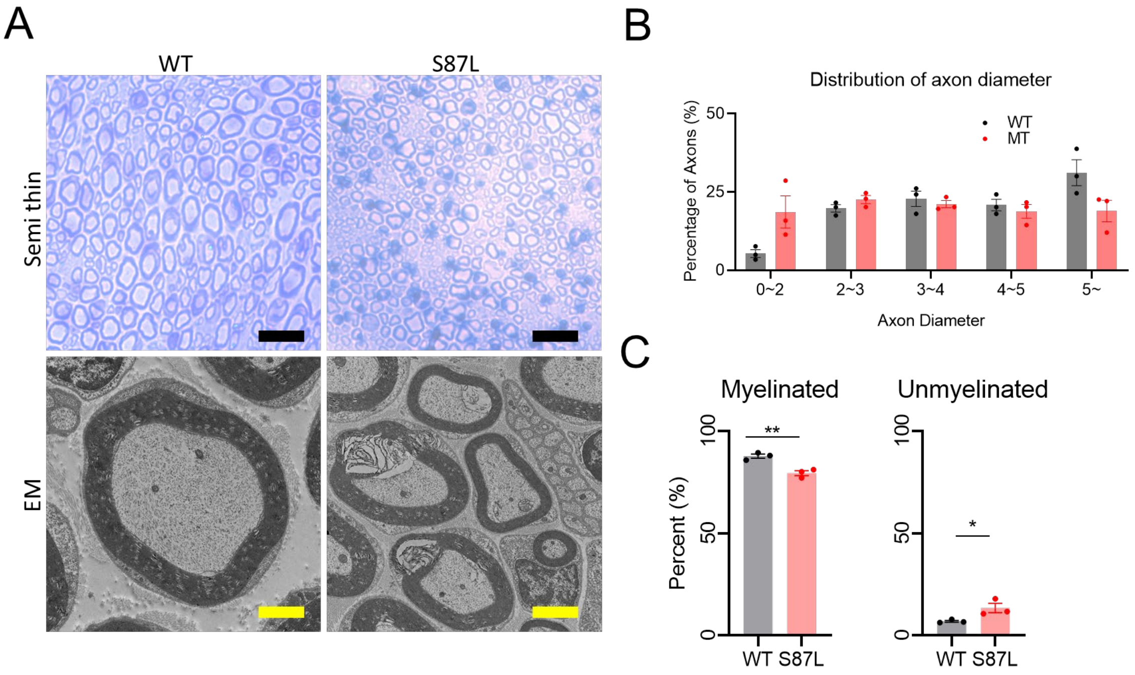

Fig. S1. Histopathological findings of 4-month-old B6.*MORC2a* S87L/+ mice. (A) Representative semi-thin cross-section images stained with toluidine blue (top) and ultra-thin electron micrographs from the same sections (bottom) from the sciatic nerves of WT and B6.*MORC2a* S87L/+ mice. Scale bar, 20 μm for semi-thin and 2 μm for ultra-thin sections. (B, C) Neuroanatomy of myelinated and unmyelinated fibers was analyzed according to genotype and nerve diameter. The dot indicates data from each sample, and data are displayed as the mean ± SEM. Statistical analysis was performed using Student's *t*-test. * $P < 0.05$, ** $P < 0.01$.

Disease Models & Mechanisms • Supplementary information

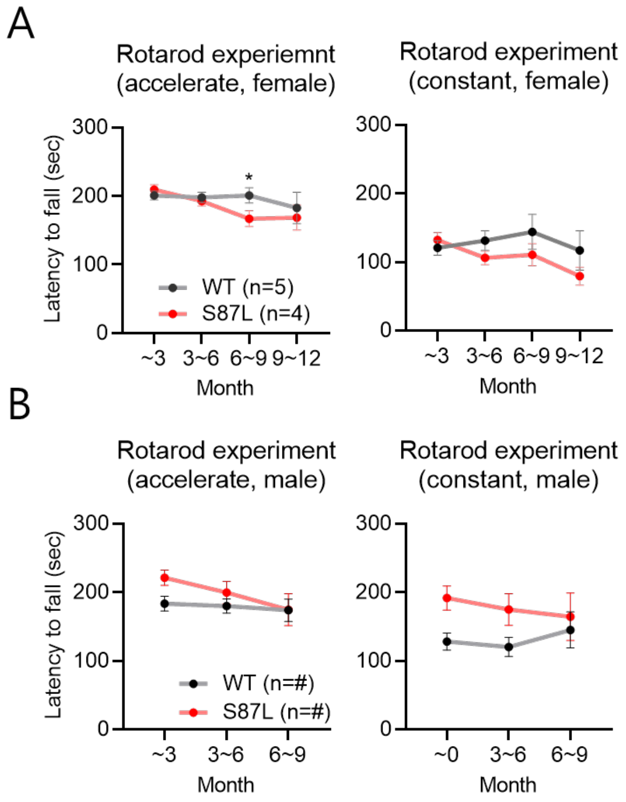

Fig. S2. Locomotive dysfunction in female B6.*MORC2a* S87/+ mice. (A, B) Analysis of the locomotive function of male (WT: n = 4; S87L: n = 3) and female (WT: n = 5; S87L: n = 4) B6.*MORC2a* S87L/+ and WT mice over a span of 12 months. The latency to fall off the rotarod was recorded in accelerating (4–45 rpm) and constant (4–30rpm) conditions. Each dot represents the data collected over 3 months (mean ± SEM). * indicates statistical significance (*P* < 0.05).

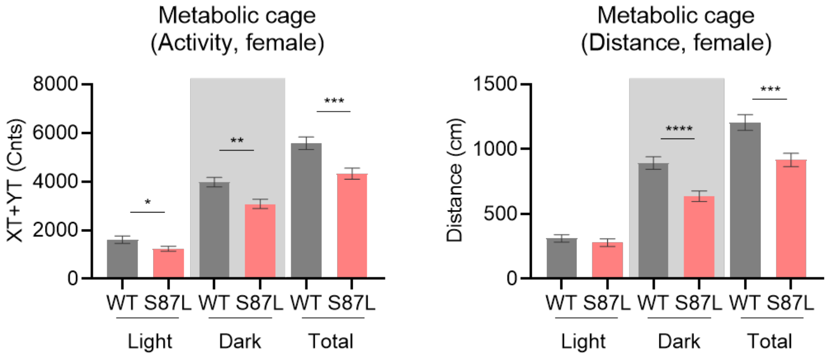

Fig. S3. *MORC2a* S87L mice exhibit low moving activity. Activity and distance of movement were analyzed for 2 days in 15-month-old female mice (WT: n = 5; S87L: n = 5). Data are the mean ± SEM. Statistical analysis was performed using Student's *t*-test. * $P < 0.05$, ** $P < 0.001$, **** $P < 0.00001$.

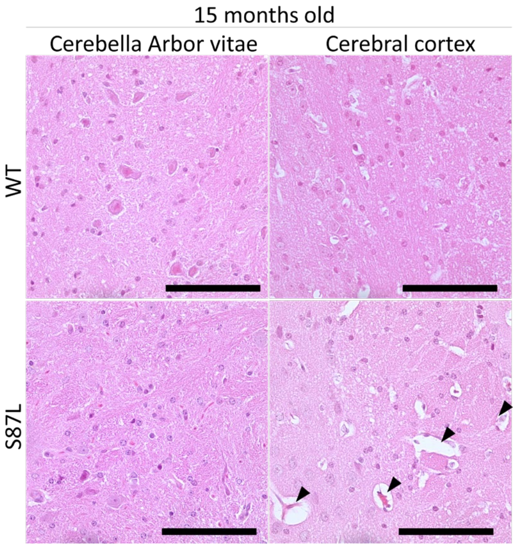

Fig. S4. *MORC2a* S87L causes neuropathy in the cerebral cortex. H&E staining was conducted using the cerebellum and cerebrum cortex of 15 months WT and B6.*MORC2a*.S87L. Black triangle point to motor neuron degeneration (Scale bar, 100µm).

Disease Models & Mechanisms • Supplementary information

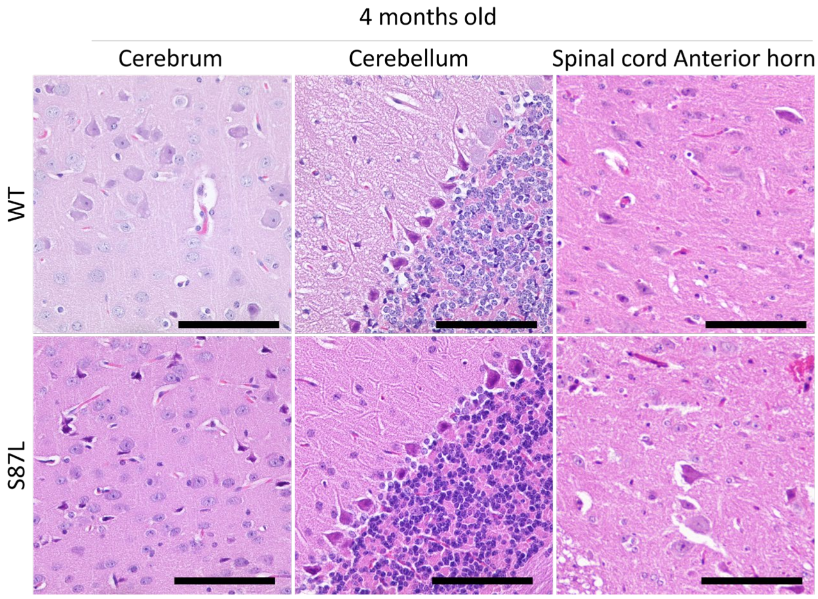

Fig. S5. **Less clinical symptoms of neuropathy in B6.*MORC2a* S87L.** H&E staining was conducted using the cerebellum and cerebrum cortex of 4 months WT and B6.*MORC2a*.S87L (Scale bar, 100µm).

Disease Models & Mechanisms • Supplementary information

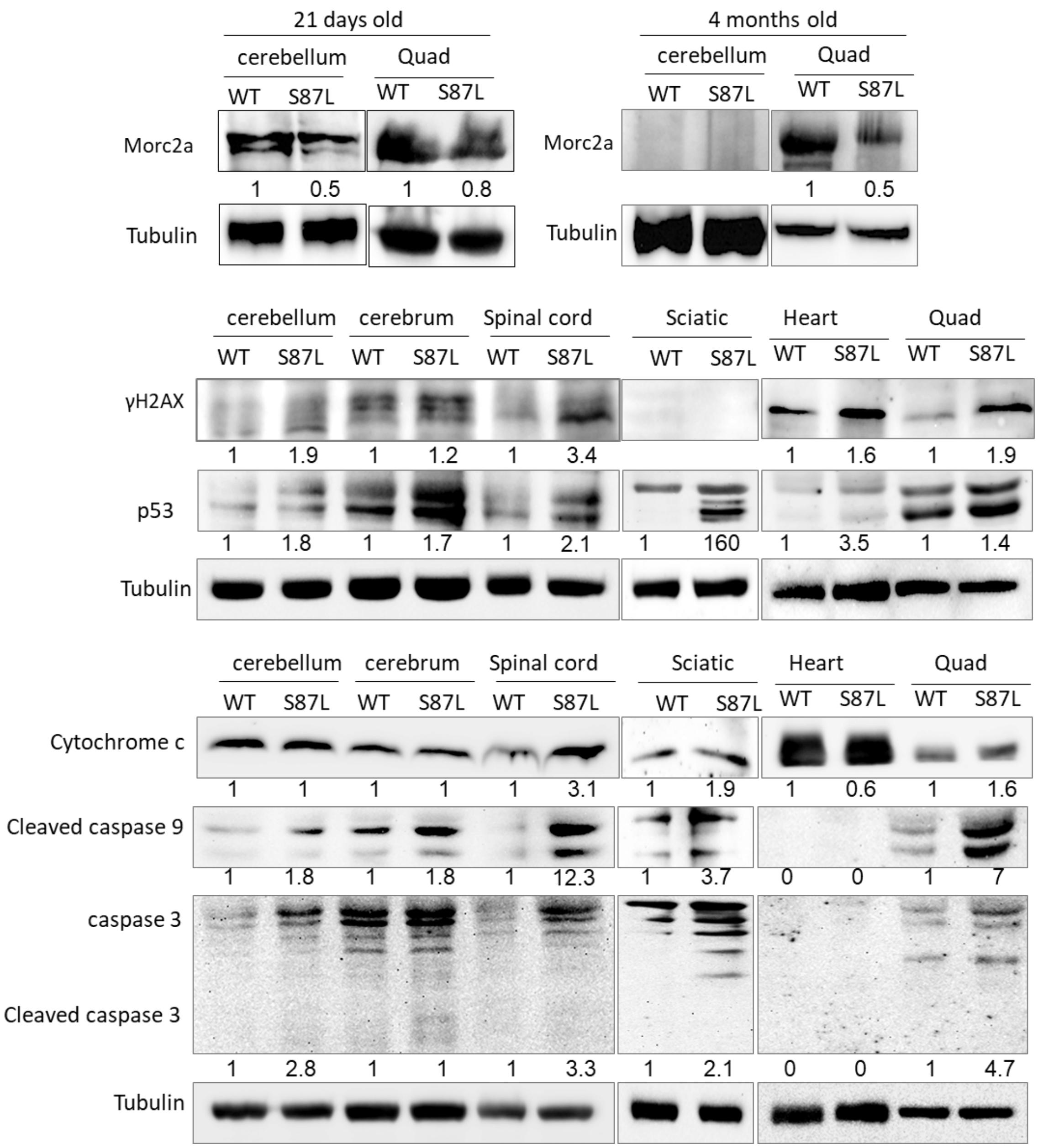

Fig. S6. **Signal quantification for each western blotting image.** Signal intensity was calculated using the Image J program. Next, signal intensities were normalized that of Tubulin, and relative intensity of WT and S87L in the same tissue were shown as a number.

Disease Models & Mechanisms • Supplementary information

Table S1. Sequences of the sgRNA and primers used in this study

	Target gene	sequence (5′→3′)	Size	Primer bank ID
sgRNA	*MORC2a*	AGTGGACTCAGGAGTTCTTTTGG		
		TTTTGGCTGACTTCCCAAACTGG		
Primer	*MORC2a*-KI	F: TGTGTTTGCCAGGTCTTGT	398 bp	
		R: GGCGTCCTCTTCGCTAAT		
	MORC2a-WT	F: AAGTCAGCCAAAAGAACTCC	321 bp	
		R: CCACTCACACCCTCAACTACA		
	MORC2a-qPCR	F: TGGAATGCTCGAACACGGG	198 bp	226529981c3
		R: GTCTAGTTCCGGCTCTCCATTA		

Table S2. Antibodies used in this study

	Target	Clone	Host	Dilution	Company (Cat number)
Primary antibodies	Morc2a	Polyclonal	Rabbit	1:1000 (WB)	Abnova (PAB15729)
	γH2AX	Polyclonal	Rabbit	1:3000 (WB)	Abcam(ab11174)
	Cytochrome-c	Polyclonal	Rabbit	1:1000 (WB)	Cell Signaling(#4272)
	Cleaved Caspase-9	Asp353	Rabbit	1:1000 (WB)	Cell Signaling(#9509)
	Cleaved Caspase-3	Asp175	Rabbit	1:1000 (WB)	Cell Signaling(#9661)
	P53	FL-393	Rabbit	1:100 (IHC)	Santa Cruz(sc-6243)
		1C12	Mouse	1:2000 (WB)	Cell Signaling(#2524)
	α-tubulin	11H10	Rabbit	1:3000 (WB)	Cell Signaling(#2125)
Secondary antibodies	α-Rabbit (biotinylated)		Goat		Vector(BA-1000)
	α-Rabbit (HRP)		Goat		AbFRONTIER(LF-SA8002)
	α-Mouse (HRP)		Goat		AbFRONTIER(LF-SA8001)
